# Bridging the gap between evidence and practice: introducing hospital-based health technology assessment in Ukraine

**DOI:** 10.1017/S0266462325103280

**Published:** 2025-11-21

**Authors:** Olena Filiniuk, Rabia Sucu, Rebecca Kohler, Viktoriia Nikulina, Kostyantin Kosyachenko, Laura Sampietro-Colom

**Affiliations:** 1 Management Sciences for Health, Ukraine; 2 Management Sciences for Health, USA; 3 Bogomolets National Medical University, Ukraine; 4 Clínic Barcelona University Hospital, Spain

**Keywords:** health technology, hospital-based health technology assessment, evidence-based approach, decision-making, healthcare facilities

## Abstract

**Objectives:**

The objective of this study is to evaluate the feasibility of implementing Hospital-Based Health Technology Assessment (HB-HTA) within Ukraine’s healthcare system, focusing on its potential to strengthen evidence-based decision-making regarding clinical effectiveness, safety, cost-efficiency, and organizational and strategic impact of health technologies (HTs) in healthcare facilities (HCFs).

**Methods:**

We collected and synthesized key initiatives implemented between 2021 and 2025, outlining the main steps involved in introducing HB-HTA in Ukraine.

**Results:**

The article describes the landscape of the Ukrainian healthcare system and shares the experience of the initial steps of HB-HTA introduction amid tight budgets and increasing demands. Drawing on international models and adapting them to the national context, we outline key implementation strategies, the development of scientific and methodological approaches for HB-HTA, and the integration of a pilot HB-HTA project within a leading national HCF known for its high-level diagnostic and operational capabilities.

**Conclusions:**

The conducted pilot laid the groundwork for integrating HB-HTA into Ukraine’s healthcare system and demonstrated its role in empowering HCFs to guide smarter budget allocation and procurement decisions, especially in the context of decentralization. With continued investment in expertise, legal integration, and streamlined methodology, HB-HTA can become a cornerstone of transparent, cost-effective decision-making across the HCFs of the National healthcare system. The experience gained in Ukraine provides valuable insights that can support other countries in effectively adopting and utilizing HTs at the hospital level.

## Introduction

The development of Health Technology Assessment (HTA) in Ukraine began many years ago and was officially institutionalized with the establishment of the HTA Department under the State Expert Center (SEC) of the Ministry of Health (MoH) in 2019 (1). On 23 December 2020, the Cabinet of Ministers of Ukraine (CMU) approved Decree Number 1300, “On the Approval of the Procedure for Conducting State Health Technology Assessment,” making HTA mandatory for all medicines covered by public funds (2). While HTA has been implemented at the national level, no regulations have been established on how HTA principles should be applied at the local level (3).

Healthcare facilities (HCFs) play a key role in utilizing health technologies (HTs) and serve as primary entry points for these advancements. According to CMU Decree Number 1300, “On the Approval of the Procedure for Conducting State HTA,” the HT is a method, procedure, system, or means for prevention, diagnosis, treatment, or medical rehabilitation, including medicines (such as medical immunobiological preparations), medical devices (such as auxiliary means for them), procedures, and organizational systems used in the field of health care (2). The rapid expansion of new and innovative HTs, such as telemedicine, robotic surgery, and artificial intelligence, holds significant potential for enhancing patient outcomes and improving healthcare efficiency. Ensuring the effective adoption of these HT, particularly in resource-limited healthcare settings, highlights the need for hospital-based HTA (HB-HTA).

HB-HTA provides a structured approach to assess HTs within the hospital context, generating evidence-based insights into managerial decision-making and optimizing healthcare delivery. Unlike national HTA, which focuses on medicines with system-wide policy implications, HB-HTA targets hospital-level assessments of medical devices, equipment, and organizational systems, tailored to local needs and resources. Although HB-HTA and national HTA have distinct scopes, they are complementary rather than redundant. This synergy reduces the burden on national HTA bodies by decentralizing routine or lower-impact assessments, enhancing efficiency, and accelerating decision-making across the healthcare system (4;5).

## Healthcare system in Ukraine: Decentralized management and centralized oversight

In October 2017, Law of Ukraine Number 2168-VIII, “On state financial guarantees for medical care for the population,” was adopted, which marked a pivotal step in the decentralization of Ukraine’s healthcare system by shifting funding from centralized budgets to a patient-centered model based on guaranteed state-financed medical services (6).

Ukraine’s healthcare system operates as a decentralized model, with certain centralized elements retained. While the system is decentralized in service delivery and hospital management, it maintains centralized control over policymaking, strategic funding, and centralized procurement. The decentralization process, particularly since the healthcare reforms of 2017, has been instrumental in shaping the current structure. HCFs are largely managed at the municipal and regional levels, with local governments (regions, cities, and municipalities). Many hospitals have transitioned into nonprofit enterprises, granting them greater financial and managerial independence, and allowing for more localized decision-making and operational flexibility (7). The National Health Service of Ukraine (NHSU), established in 2018, contracts healthcare providers directly, enabling facilities to receive funding based on services delivered rather than a centralized allocation model (8).

In this decentralized system, the MoH maintains responsibility for establishing national healthcare policies, regulatory frameworks, and clinical guidelines, ensuring uniformity across the country. State funding for specialized care, such as NHSU’s State Reimbursement Program, which covers outpatient prescriptions for chronic conditions, is centrally controlled. Furthermore, the Medical Procurement of Ukraine (MPU) oversees the centralized purchase of medicines and medical equipment for certain state programs, ensuring coordinated procurement and distribution throughout the country.

To strengthen the resilience of the healthcare system and optimize resource utilization, the restructuring of the hospital network was launched in 2022 (9). This reform aligns with the government’s healthcare decentralization policy, addressing contemporary challenges and enhancing system efficiency that was enacted in Law of Ukraine Number 2801-XII, dated 19 November 1992, and reviewed on 27 March 2025 – “Fundamentals of Ukrainian legislation on healthcare” (10).

Within the framework of ongoing decentralization reforms in Ukraine’s healthcare system, which aim to transfer greater autonomy and decision-making authority to local governments and HCFs to improve responsiveness and efficiency, a newly introduced capable network is designed to ensure the rational allocation of resources, meet population healthcare needs, and integrate technological and professional capacities for delivering more advanced healthcare services. The clustering process was based on a comprehensive assessment of key factors, including administrative-territorial boundaries, regional geography and road infrastructure, demographic trends, morbidity and mortality patterns, optimal patient referral pathways, and the workload of healthcare professionals.

Based on the range of healthcare services provided, a capable network comprises three types of hospitals: supercluster hospitals, which deliver highly specialized and complex care; cluster hospitals, which provide general hospital services for a population of ~150,000; and general hospitals, which offer basic healthcare services for populations ranging from 50,000 to 80,000 (11).

**Figure 1.**
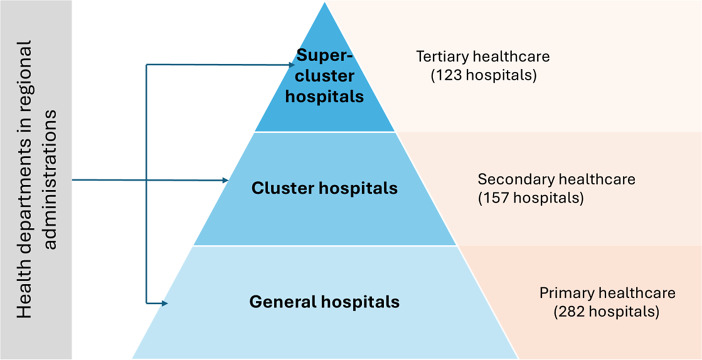
Capable network in the Ukrainian healthcare system.

As of 6 April 2024, the institutions of the capable network included 562 HCFs across 19 regions, categorized as follows: 123 superclusters, 157 clusters, and 282 general hospitals (12). It is important to note that cluster and supercluster facilities are hospitals with a high level of annual implementation of new HTs. The structure of the capable network within the Ukrainian healthcare system is illustrated in Figure 1.

The operating budgets of HCFs are derived from various funding sources. The Program of Medical Guarantees (PMG) under the NHSU covers essential HCF expenditures, including personnel costs, medical supplies, pharmaceuticals, and critical healthcare services provided to the population. In 2025, the PMG budget amounted to ~175.5 billion UAH, equivalent to about 4.22 billion USD, based on an average exchange rate of 41.58 UAH per USD (13). In addition to PMG funding, HCFs may receive financial support from regional or local government authorities, private sector contributions, patient co-payments, and external sources such as international donors or partnerships. Also, it is important to notice that the full-scale war has played a critical role in dramatically increasing the flow of direct funding to hospitals, including resources for reconstruction, capacity building, specialized equipment, and essential medicines. These additional funds have significantly strengthened HCF functionality. This diverse funding structure plays a crucial role in shaping the financial sustainability and operational efficiency of HCFs. As healthcare systems evolve and decision-making processes become increasingly data-driven, the introduction of HB-HTA can provide a robust, evidence-based framework to support informed investment and disinvestment decisions by regional and local authorities, while also influencing central-level prioritization for centralized procurement. The HB-HTA approach can act as a transparent, unified tool for optimizing resource utilization and mitigating corruption, fostering greater accountability in healthcare spending.

## Introducing HB-HTA in Ukraine

### Setting the grounds

As Ukraine advances its healthcare reforms, including the decentralization of healthcare services and the strengthening of HTA practices, the implementation of HB-HTA has become a pivotal step toward evidence-based decision-making, ensuring the efficient allocation of resources within HCFs.

The introduction of HB-HTA in Ukraine began with several awareness-raising activities aimed at engaging diverse stakeholders and fostering a shared understanding of its benefits to hospital managers and patients. In 2021, PhD research was initiated at the Bogomolets National Medical University, focusing on the development of scientific and methodological approaches for implementing HB-HTA in the Ukrainian healthcare system (14). The study started by exploring the existing regulatory framework for the introduction of HTs in HCFs. Through a comprehensive evaluation of the current regulatory framework of HT implementation, specifically medicines and medical devices, several aspects were identified. First, the selection process for medicines purchased outside the National Essential Medicines List remains unregulated, which leads to inconsistencies in procurement decisions, potentially affecting equitable access to cost-effective treatments. Second, national legislation governing medical devices does not align with European Union (EU) standards. This misalignment creates challenges in harmonizing regulatory requirements, ensuring product safety, and facilitating international integration. Finally, the absence of a standardized regulatory framework for evaluating and selecting HTs at the local level, coupled with the lack of clear regulatory mechanisms, hinders evidence-based decision-making and efficient resource allocation within HCFs (3).

Furthermore, from Quarter 3 (Q3) 2022 to Q1 2023, a series of semi-structured interviews with hospital managers was carried out to examine the current practices and approaches for the introduction of HTs in Ukraine (15). Twenty-eight respondents from three HCFs across different regions of Ukraine took part in the online semi-structured interviews, each lasting between 1 and 1.5 h. The HCFs were selected based on several criteria: high levels of diagnostic and surgical activity, representation from different regions of Ukraine, and strong support from the general management, reflecting the critical role of managers in driving successful innovation implementation. Moreover, the respondents were chosen for their involvement in the decision-making process regarding the implementation of new and innovative HT.

This research revealed a lack of familiarity with the HTA concept among hospital managers, leading to significant follow-up consequences. In Ukrainian HCFs, while the procurement of HTs is well-regulated under the Law of Ukraine “On Public Procurement,” there are no specific recommendations, guidelines, or standardized procedures for the pre-procurement stage. The process varies across HCFs, involving different stages, timelines, and levels of stakeholder engagement. Furthermore, there are no integrated procedures for making decisions related to HT disinvestment in any of the facilities surveyed.

Currently, decision-making around which HTs to purchase relies on limited sources of information, lacking comprehensive literature reviews or robust comparative analyses with existing practices or placebo-controlled evaluations. This gap significantly impairs managers’ ability to make well-informed choices that consider all relevant clinical, economic, organizational, and strategic aspects when assessing the appropriateness of a specific HT for a given hospital. Addressing these gaps through structured methodologies and evidence-based frameworks is essential for optimizing HT management and decision-making in hospitals.

One of the focuses of this study was identifying specific challenges for the future implementation of HB-HTA in Ukraine. Respondents highlighted several critical barriers, including the need to foster strong leadership support at the local level for HTA implementation, provided by heads of HCFs, regional authorities, or both, secure sufficient investment in developing HTA expertise within HCFs, provide comprehensive professional training for staff, and establish robust data collection and management systems. Addressing these challenges is essential for the successful implementation of HB-HTA, ensuring its sustainability and effectiveness in improving decision-making on the HCF level.

This study showed the potential benefits of implementing HB-HTA in the examined hospitals, emphasizing the need for structured HT assessment methodologies, evidence-based selection criteria, and integrated disinvestment frameworks to optimize HT management in Ukrainian HCFs.

### Adapting and transferring HB-HTA knowledge to professionals

Given the significant knowledge gap in HTA in Ukraine, researchers from the Bogomolets National Medical University designed an HB-HTA training methodology tailored for hospital stakeholders involved in decision-making regarding the adoption of HTs (16). The curriculum covered key HB-HTA aspects and was structured into eight modules, each lasting 1.5 hours. Each module combined theoretical and practical components and concluded with an assignment, which was reviewed and discussed at the beginning of the following session. This interactive approach facilitated better knowledge retention and practical application of concepts.

From September to November 2022, the training methodology was piloted at Zhytomyr Regional Clinical Hospital named after O. F. Herbachevsky. Nine hospital stakeholders participated, including the Medical Director of Therapy, the Medical Director of Surgery, the Deputy Director for Economic Affairs, the Head of the Monitoring Department, representatives from the Information and Statistics Department, two clinical departments, the Laboratory Department, and the Functional Diagnostics Department. To evaluate the effectiveness of the training, a post-training knowledge assessment was conducted, revealing that participants demonstrated average to above-average levels of knowledge assimilation. The piloted curriculum continues to be used for HB-HTA training hospital managers across various HCFs in Ukraine.

A key aspect of implementing HB-HTA in Ukraine involved a comprehensive review of global HB-HTA methodologies to identify the most suitable ones for the Ukrainian healthcare system. Currently, HB-HTA frameworks are well-established in Canada, the United States, Australia, Asia, the United Arab Emirates, and numerous European countries. As a priority for adaptation in Ukraine, the AdHopHTA (Adopting Hospital-based Health Technology Assessment) methodology was selected (17). This methodology was developed by a group of experts from multiple countries, including Norway, Finland, Turkey, Spain, Austria, Italy, Estonia, Denmark, and Switzerland, under the leadership of the Clinic Barcelona University Hospital. To tailor the AdHopHTA methodology to the Ukrainian context, SAFEMed supported the organization of an adaptation workshop with stakeholders from nine HCFs (18) ensuring diverse regional representation and coverage across all levels of the healthcare system (macro, meso, and micro). The key value of this workshop lay in the expert insights shared by the participants, whose knowledge was carefully analyzed and synthesized into adapted Ukrainian HB-HTA tools. This complex adaptation process, which combined an online discussion with follow-up correspondence via email, ensured the precise customization of AdHopHTA methodologies to the Ukrainian healthcare context.

A fundamental strategic step was the development of comprehensive methodological recommendations for implementing HB-HTA in HCFs, created by a group of experts from Bogomolets National Medical University (19). These recommendations synthesize international experience in HB-HTA implementation, incorporating key insights from AdHopHTA (17) and Danish Centre for Evaluation and Health Technology Assessment (20), along with feedback from hospital stakeholders gathered during in-depth interviews and the curriculum piloting process. Drawing on best practices and global frameworks, the guidelines provide both theoretical foundations and practical tools, offering hospitals actionable strategies for effectively conducting HB-HTA at the appropriate level and ensuring its smooth integration into hospital systems. In 2023, these methodological recommendations were finalized, officially approved by Bogomolets National Medical University, and disseminated across HCFs in Ukraine.

### Piloting HB-HTA in practice

As a logical next step, key national stakeholders, with SAFEMed technical support, began implementing an HB-HTA pilot project as a way to equip stakeholders and HCF personnel with essential knowledge, methodologies, and practical tools in the application of HB-HTA in the Ukrainian context. To select a pilot hospital, a core team of researchers from Bogomolets National Medical University evaluated a set of hospitals participating in the adaptation methodology process based on predefined inclusion and exclusion criteria (Table 1).

**Table 1.** Inclusion and exclusion criteria

Inclusion criteria	Exclusion criteria
Participation in the HB-HTA adaptation workshop	Hospitals are already engaged in another pilot project
Strong operational and diagnostic capacity, demonstrating the consistent application of HTs in practice	Hospitals located in frontline areas
High managerial interest in implementing new HTs	Hospitals with no interest or willingness to pilot HB-HTA
	Hospitals where piloting would be challenging to scale across different levels of healthcare

As a result, the team identified the Amosov National Institute of Cardiovascular Surgery (the Institute) as a prime candidate for the HB-HTA pilot project. This HCF demonstrated a strong commitment to participation, possesses advanced diagnostic and operational capacities, and offers significant potential for scaling and disseminating pilot outcomes to hospitals across different levels of the healthcare system. The Institute is a leading center for cardiovascular surgery in Ukraine, with a unique experience in performing complex surgical interventions. It is equipped with state-of-the-art, innovative HTs that enable the execution of the most intricate surgeries at the highest level. The Institute not only provides high-quality medical services but also actively engages in scientific research in the field of cardiovascular surgery (21).

The pilot project was implemented within the framework of the Memorandum of Cooperation between the Institute, the SEC of the MoH, the Bogomolets National Medical University, and the international organization Management Sciences for Health, Inc., through the SAFEMed project.

A roadmap was developed to structure the process, enhance transparency, ensure accountability, and optimize resources for implementing the pilot project. The process consisted of five key stages: forming a multidisciplinary team, ensuring the educational component, selecting a potential HT for HB-HTA, conducting the HTA, and making managerial decisions.

To facilitate effective implementation of the pilot, the Institute assembled a multidisciplinary team (the Institute’s working group) of administrative, clinical, and economic experts with clearly defined roles and responsibilities. The organizational structure was designed as an independent group, with members contributing voluntarily and not dedicated full-time to HTA activities (17). Following the project’s kick-off meeting, the team’s composition was formally approved by an Institute order, based on predefined selection criteria, ensuring alignment with the pilot’s objectives.

The pilot HB-HTA was based on the developed recommendations for implementing HB-HTA in healthcare institutions. To enhance the capacity of the selected working group, a comprehensive training session was conducted to introduce the key aspects of HB-HTA. During the initial training, the working group identified and prioritized HT for assessment, focusing on the comparison of aortic valves with skirt versus without skirt for transcatheter aortic valve implantation (TAVI) in patients with aortic valve stenosis.

The introduction of skirted valves at the Institute represents a strategic advancement aimed at improving clinical outcomes and optimizing resource utilization. A comprehensive evaluation grounded in the main aspects of HB-HTA was conducted. Based on the findings, skirted valves were recommended as the preferred option for TAVI at the Institute. Their adoption is projected to improve patient outcomes by reducing complications and repeat interventions while generating significant cost savings in the long term. These efficiencies will alleviate financial pressures on the Institute’s budget, streamline departmental workflows, and enhance the overall allocation of healthcare resources.

The results of the pilot project were presented at a Round Table discussion in December 2024, attended by participants who signed the Memorandum, as well as representatives from the SEC, NHSU, MPU, and other Institutes of the National Academy of Medical Sciences of Ukraine (22). During the Round Table, the MPU expressed a keen interest in the results of the Pilot Project, recognizing its potential to inform future procurement-related decision-making at the central level.

It was widely acknowledged that, given the successful implementation of the pilot program within the National Medical Academy of Sciences’ specific institutional framework, expanding the pilot to include other hospital types, such as sub-cluster and cluster facilities, is essential. Broadening the scope of this initiative and disseminating the results across HCFs nationwide will be critical to ensuring its scalability and impact.

**Figure 2.**
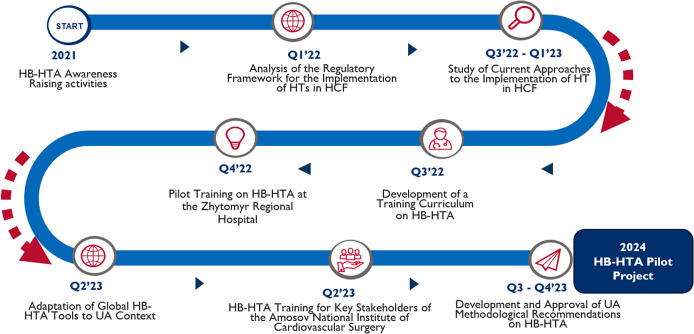
Execution framework for implementing HB-HTA in Ukraine.

The comprehensive Execution Framework for implementing HB-HTA in Ukraine is outlined in Figure 2.

## Implications and areas for further study

The implementation of HB-HTA in Ukraine offers significant potential; however, several challenges must be addressed to ensure its effectiveness. This section applies a SWOT analysis framework to identify the key strengths, weaknesses, opportunities, and threats that should be carefully considered to support the successful scale-up of HB-HTA in the country (Table 2).

**Table 2.** SWOT analysis of HB-HTA implementation in Ukraine

Strengths	Weaknesses
Ongoing decentralization of the healthcare system, enabling greater autonomy in decision-making. Independent budget allocation by HCFs, allowing for tailored resource management. Urgent need for disinvestment mechanisms for outdated HTs, creating momentum for HB-HTA implementation. Government of Ukraine (GOU) prioritizes HTA development, ensuring policy support and alignment. An established scientific and methodological foundation for implementing HB-HTA in the Ukrainian healthcare system. Strong expertise within the HTA department of the SEC, providing a solid foundation for HB-HTA expansion and technical guidance.	Limited HTA expertise within HCF, hindering effective adoption and integration. Insufficient national and regional level leadership support, impacting the prioritization and sustainability of HB-HTA initiatives. Inadequate data collection and management systems, affecting the quality and accessibility of evidence for decision-making. Weak identification of unmet needs and limited recognition of how HB-HTA can address challenges within HCF. Lack of coordination and support from the HTA department, undermining the strategic alignment and effectiveness of HB-HTA efforts.

Opportunities	Threats
Dissemination of professional training programs to hospital management to enhance HTA capabilities. Incorporation of the “Hospital-based HTA” term into Ukrainian legislation, providing a formal framework for its implementation. Expanding best practices and standardization of methodology, with the development and submission of National HB-HTA guidelines. Professional development for HCF management and fostering international collaboration via HTA networks. Enhancement of HCF autonomy with the implementation of a regional MEA that can consider HB-HTA recommendations during decision-making processes.	Limited resources for the healthcare system. Insufficient transfer of HB-HTA results to practical implementation. Bias among stakeholders and lack of transparency in procurement processes. Low quality of acquired results due to the use of nonstandardized methodologies. Impact of ongoing full-scale war and uncertainty, which may hinder or delay the development and implementation of HB-HTA in the country.

The identified strengths arise from the broader national context, where the need for the rational use of public funds has become increasingly pressing. In the framework of ongoing decentralization reforms and a strong political commitment to HTA, the Government of Ukraine has established a solid foundation for HTA development by mandating its use for all publicly funded medicines. This policy shift has created an enabling environment for evidence-based decision-making. Moreover, the HTA Department of the SEC offers a high level of technical expertise, which serves as a valuable resource to further advance the implementation and institutionalization of HTA practices across all levels of the healthcare system of Ukraine.

However, we observe the limited availability of HTA graduate and postgraduate programs in Ukraine, which restricts the development of HTA expertise and capacity at the HCF level. This, coupled with insufficient leadership support, constitutes a major concern within the weaknesses during HB-HTA implementation. Other challenges include the lack of robust data collection mechanisms in HCF, which limits the availability of reliable evidence for decision-making. Coordination between national and local HTA levels still requires strengthening, including establishing a centralized database to facilitate the effective dissemination and use of HB-HTA results. Furthermore, the limited understanding of how the HB-HTA tool can enhance decision-making at the HCF level constrains its development and wider adoption.

Opportunities for the development of HB-HTA in Ukraine have also been identified. A key step is the formal introduction of the term “Hospital-based HTA” into Ukrainian legislation, which would provide a legal foundation for its systematic implementation. Additionally, the growing awareness of HB-HTA and the expansion of expert capacity in this area offer potential for professional development and international collaboration through HTA networks. Further opportunities include the standardization of methodologies and the development of National HB-HTA guidelines, which would align healthcare policy with the best internal and international practices. Finally, it enhances the autonomy of HCFs, particularly through the implementation of regional managed entry agreements (MEA) that can consider HB-HTA recommendations during decision-making processes.

The development of HB-HTA in Ukraine may encounter threats associated with limited healthcare financing brought about by the full scale, which has already placed deep and lasting impacts on the health system. Additionally, the risk of stakeholder bias and nontransparent procurement practices remains a concern and must be carefully monitored. Another important issue is the insufficient translation of HB-HTA results into practical implementation, which may discourage HCFs from adopting and sustaining the HB-HTA approach.

In the study by Lipska et al., experts identified a key external barrier to HB-HTA as the absence of formal acknowledgment in national or regional legislation, while internally, a major challenge was the lack of support from top hospital management (23). Similarly, in Ukraine, while national-level HTA has gained legal and institutional support, the integration of HB-HTA at the hospital level faces comparable obstacles – there is no formalized legal framework specifically supporting HB-HTA, and many hospitals lack support from top management to implement such assessments effectively.

## Conclusion

HB-HTA is a valuable tool for hospital managers, facilitating evidence-informed decision-making in the adoption of HT while providing a degree of legal protection. Although significant progress has been made in introducing HB-HTA in Ukraine and achieving the initial objectives of the implementation framework, its nationwide uptake remains limited.

The establishment of a legal framework, specifically, the inclusion of the term “Hospital-Based HTA” in Ukrainian legislation, alongside the national approval of comprehensive HB-HTA guidelines, would create favorable conditions for the HB-HTA tool’s wider implementation across HCF.

Moreover, the potential introduction of a regional MEA that incorporates HB-HTA recommendations into decision-making processes would further promote its systematic dissemination and integration into routine hospital-level governance. This advancement would provide a robust legal foundation for the consistent application of HB-HTA across all levels of healthcare delivery.

Sustained policy commitment, enhanced capacity building, and strengthened institutional coordination remain essential to establish HB-HTA as a permanent and effective element within Ukraine’s healthcare decision-making ecosystem.

The Ukrainian experience offers valuable insights for other countries seeking to strengthen hospital-level decision-making. Key lessons include the importance of selecting hospitals with high diagnostic and operational activity to maximize clinical and budgetary impact; securing strong managerial support to ensure the integration of assessment outcomes are integrated into hospital decision-making; and allocating dedicated time for working group members, as HB-HTA requires structured and continuous effort beyond routine clinical duties. Moreover, capacity building and targeted training are crucial, especially at HCF, where HTA expertise remains limited.

Collectively, these findings demonstrate that HB-HTA can be effectively implemented in other countries, even in resource-constrained settings, providing a practical pathway to strengthen evidence-based decision-making and optimize the adoption and utilization of HTs at the hospital level.
